# Preliminary results in quantitation of HLA-DRA by real-time PCR: a promising approach to identify immunosuppression in sepsis

**DOI:** 10.1186/cc13046

**Published:** 2013-10-06

**Authors:** Sara Cajander, Anders Bäckman, Elisabet Tina, Kristoffer Strålin, Bo Söderquist, Jan Källman

**Affiliations:** 1 Department of Infectious Diseases, Orebro University Hospital, Sodra Grev Rosengatan, 70362, Orebro, Sweden; 2 School of Health and Medical Sciences, Orebro University, Faktultetsgatan 1, 70218, Orebro, Sweden; 3 Clinical Research Centre, Orebro University Hospital, Sodra Grev Rosengatan, Orebro, Sweden; 4 Department of Infectious Diseases, Karolinska University Hospital, 141 86, Huddinge, Sweden; 5 Department of Laboratory Medicine, Clinical Microbiology, Sodra Grev Rosengatan, Orebro University Hospital, Orebro, Sweden; 6 Faculty of Medicine and Health, Orebro University, Fakultetsgatan 1, 70218, Orebro, Sweden

## Abstract

**Introduction:**

Reduced monocyte human leukocyte antigen (mHLA)-DR surface expression in the late phase of sepsis is postulated as a general biomarker of sepsis-induced immunosuppression and an independent predictor of nosocomial infections.

However, traditional monitoring of mHLA-DR by flow cytometry has disadvantages due to specific laboratory requirements. An mRNA-based HLA-DR monitoring by polymerase chain reaction (PCR) would improve the clinical usage and facilitate conduction of large multicenter studies. In this study, we evaluated an mRNA-based HLA-DR monitoring by quantitative real-time PCR (qRT-PCR) as an alternative method to traditional flow cytometry.

**Methods:**

Fifty-nine patients with sepsis and blood culture growing pathogenic bacteria were studied. Blood samples were collected at day 1 or 2 after admission, for measurement of mHLA-DR by flow cytometry and mRNA expression of HLA-DRA and class II transactivator (CIITA) by qRT-PCR. Blood samples from blood donors were used as controls (n = 30).

**Results:**

A significant reduced expression of mHLA-DR, HLA-DRA, and CIITA was seen in septic patients compared with controls*.* HLA-DRA mRNA level in whole blood was highly correlated with surface expression of mHLA-DR.

**Conclusions:**

Patients with sepsis display a diminished expression of HLA-DR at the monocyte surface as well as in the gene expression at the mRNA level. The mRNA expression level of HLA-DRA monitored by qRT-PCR correlates highly with surface expression of HLA-DR and appears to be a possible future biomarker for evaluation of immunosuppression in sepsis.

## Introduction

Septic syndromes caused by bloodstream infections represent a major health-care problem worldwide [1], and sepsis is the leading cause of mortality in non-cardiac intensive care units (ICUs) [2]. The mortality rate of severe sepsis remains high (approximately 30%) despite improved clinical management algorithms [3]. In a recent retrospective study of the outcome in 999 patients with severe sepsis, the overall mortality was 31%, and the highest incidence of deaths (67.3%) was in the late phase [4].

The initial phase of severe sepsis is often characterized by an intense hyperinflammation with massive release of proinflammatory cytokines. For many years, this “cytokine storm” was thought to be responsible for the high mortality rate and multiple organ dysfunction in septic syndromes [5,6]. However, in spite of numerous drug trials aiming to counteract the proinflammatory activation, the results have been disappointing [7-10]. In 2011, the worldwide PROWESS (Recombinant Human Activated Protein C Worldwide Evaluation in Severe Sepsis) SHOCK trial resulted in a withdrawal of recombinant protein C (Xigris; Eli Lilly and Company, Indianapolis, IN, USA), because of its demonstrated lack of clinical efficacy [11].

Many patients who survive the initial critical phase of septic shock die at a later time point due to secondary infections with pathogens normally deleterious only in immunocompromised hosts [4,12]. Thus, investigators of a new paradigm have proposed that a hypoinflammatory and immunosuppressive state plays a central role in sepsis [5,6,13-17]. A recent post-mortem study showed that patients who die in the ICU following sepsis display biochemical, flow cytometric, and immunohistochemical findings consistent with immunosuppression [18].

Immunostimulation by granulocyte-macrophage colony-stimulating factor (GM-CSF) and interferon-gamma (IFN-γ) during the state of sepsis-induced immunosuppression might be a promising therapeutic option to reverse this anergy [19,20]. However, since immunostimulants can be deleterious when given in the hyperinflammatory state of sepsis, a safe and stable biomarker of the immunologic state is crucial. Downregulation of monocyte human leukocyte antigen-DR surface expression (mHLA-DR) measured by flow cytometry is postulated as a general biomarker of sepsis-induced immunosuppression and acts as an independent predictor of nosocomial infections [21]. However, the use of mHLA-DR as a marker of immunosuppression is not yet sufficiently evaluated in large multicenter studies of patients with sepsis, and this is most likely due to pre-analytical requirements and limitations in specimen handling. Flow cytometric measurements of mHLA-DR require handling of blood samples within 4 hours [22], hindering inclusion from centers lacking flow cytometry.

A global transcriptional downregulation of a gene panel required for mHLA-DR expression has been demonstrated in whole blood from patients with sepsis [23,24]. If HLA-DR expression as a marker of immunosuppression could be monitored by quantitative real-time polymerase chain reaction (qRT-PCR) instead of traditional flow cytometry, it would facilitate future multicenter studies.

The aim of this study was to evaluate whether monitoring of HLA-DR by qRT-PCR supports previous reports describing significant reduced expression levels of HLA-DRA and class II transactivator (CIITA) in patients with sepsis [23,24] and, furthermore, to assess how it correlates with traditional analysis performed by flow cytometry.

## Materials and methods

### Patient selection

Ethical approval for the study was obtained from the Ethics Committee (Central Review Board, Uppsala, Sweden). This was a single-center study at Örebro University Hospital Sweden. The study group consisted of patients (n = 59) who had sepsis and positive blood culture growing pathogenic bacteria and who were enrolled during a 19-month period. Three patients were excluded from the 62 patients who were initially recruited for the study: two patients had bacterial findings regarded as a contamination (coagulase-negative staphylococci) and no evidence of clinical infection, and one patient did not fulfill inclusion criteria, due to delay in blood culture sampling. Sepsis severity definitions (sepsis, severe sepsis, and septic shock) were based on classic criteria defined by the American College of Chest Physicians/Society of Critical Care Medicine [25]. When criteria for sepsis, but not severe sepsis or septic shock, were met, we defined it as non-severe sepsis. Blood cultures were collected on admission day 0 from all patients who had suspected infectious disease and who were admitted to the Department of Infectious Diseases and Department of Internal Medicine. When blood cultures showed growth of pathogenic bacteria within 1 or 2 days from admission, patients were consecutively enrolled in the study. All patients were included after informed consent to participate and consent to publish. At day 1 or 2 after admission day, blood samples for both flow cytometry and mRNA-based monitoring of HLA-DRA were obtained. Blood samples from blood donors (n = 30) at the university hospital in Örebro were randomly collected and used as controls. Comorbidity of the patients with sepsis was assessed by Charlson comorbidity score [17].

### Sampling

Sterile vacuum tubes (PAXgene Blood RNA tube; PreAnalytiX GmbH, Qiagen group, Hilden, Germany) were used in the sampling of peripheral whole blood for PCR analysis. The PAXgene tubes were stored after sampling in -80°C until further analysis. EDTA anticoagulant tubes were used in the sampling of peripheral whole blood for flow cytometry analysis of HLA-DR. The samples for flow cytometry were immediately placed on ice and handled within 4 hours.

### RNA isolation

RNA was prepared by using the PAXgene Blood RNA-kit (PreAnalytiX GmbH, Qiagen group) in accordance with the instructions of the manufacturer. The concentration and purity of RNA were measured on a NanoDrop ND-1000 Spectrophotometer (Agilent Technologies Inc., Santa Clara, CA, USA) while using buffer (10 mM Tris–HCl buffer, pH 7.5) as a blank. The ratio of absorbance at 260 and 280 nm was used to assess the purity. A ratio of approximately 2.0 was accepted as pure. The RNA preparation was kept frozen (-80°C) prior to use.

### cDNA preparation

A volume corresponding to 100 ng of RNA was used in synthesis (20 μL) of cDNA by using a High-capacity cDNA reverse transcription kit (#4368814; Applied Biosystems, Foster City, CA, USA) in accordance with supplied instructions. This synthesis was performed in duplicate, and the products were pooled prior to use. A non-sample preparation was also performed as an internal control. The cDNA was stored at -80°C.

### Gene expression assays

The expression levels of mRNA encoding a non-polymorphic region of the alfa-chain of the HLA-DR molecule (HLA-DRA) and the mRNA coding for CIITA, the major regulator of the transcription of HLA-DR genes, were obtained by quantitative real-time PCR.

The cDNA (2 μL) was tested in the following TaqMan Gene expression assays (FAM-labeled MGB probes) (Applied Biosystems/Life Technologies Europe BV, Stockholm, Sweden): HLADRA-A (Lot Hs00219575_ml), RefSeq: NM-019111.4, Amplicon 97 base pairs (bp); CIITA (Lot Hs00172094_ml), RefSeq: NM_000246.3, Amplicon 57 bp; and peptidylpropylisomeras B (PPIB) (Lot Hs00168719_ml), RefSeq: NM_000942.4, Amplicon 67 bp.

These assays were run in triplicates (20-μL reactions) in a 96-well (MicroAmp fast, part #4346907) fast format (Applied Biosystems) and a relative quantification mode, using the TaqMan Universal MasterMix (Applied Biosystems, part #4352042, No AmpErase UNG) on a ABI7900HF Fast Real-Time PCR-instrument (Applied Biosystems) using the recommended two-step fast-program for 40 cycles. Nuclease-free water samples (Life Technologies Europe BV) were used as a negative control and calibrator. The resulting PCR data were controlled for errors, which were removed prior to detector-centric analysis. Samples with errors in amplification triplicates were re-run in a new plate, and analyzed in the same way. The intra-assay variation within triplicates was low: change in threshold cycle (ΔCt) standard deviation (SD) was less than 0.1.

Data from every separate plate (n = 12) were analyzed by using automatic threshold and baseline and a detector-centric mode (SDS2.3 and RQ manager 1.2; Applied Biosystems).

The resulting average ΔCt values for the samples versus the reference gene PPIB were used in calculating the fold change assuming equal efficiency for all three assays within the same sample and run. PPIB was used as reference gene because of its previously described stability in inflammatory conditions [26]. The Ct value for PPIB was an average of 25.5 (SD of 0.77) for the 12 separate PCR plates.

Before the clinical samples were run, efficiency calculations of the individual assays were performed on sample dilutions to verify similar activity of the PCR in the used gene expression assays. The compared gene assays were comparable in efficiency and over 96% for cDNA from samples and controls. The inter-assay variation of the ratios between different runs and samples (n = 14) were an average of ±14% for both genes. The differences in Ct value for a separate sample were less than 0.5 between the different runs in 13 out of 14 samples.

### Flow cytometry

The expression of cell surface HLA-DR on monocytes was assessed at day 1 or 2 after admission by standardized flow cytometry [22]. Antibody staining was performed within 4 hours after sampling by using QuantiBRITE™ Anti–HLA-DR PE*/Anti-Monocyte PerCP-Cy5.5 (BD Biosciences, San Jose, CA, USA) and QuantiBRITE™ PE* (BD Biosciences) in accordance with the instruction of the manufacturer. A FC500 (Beckman Coulter, Fullerton, CA, USA) equipped with an argon laser (488 nm) and HeNe laser (633 nm) and EXPO 32 software (Kaluza v1.2, Beckman Coulter) was used for data analysis, and results are expressed as number of antibodies bound per cell (AB/c).

### Statistical analysis

Groups were compared by the Mann–Whitney *U* test (significance level *P* <0.05). Spearman rank correlation coefficient was used for calculation of significant correlation between the two different methods. Significant correlation was set at the 0.01 level (two-tailed). Data are given as median ± interquartile range (IQR).

## Results

### Characteristics of patients with sepsis

The study group consisted of 59 patients with sepsis and concomitant bacteremia. Forty-eight patients were defined as non-severe sepsis, 8 patients as severe sepsis, and 3 patients with septic shock. Twelve patients (20%) were admitted to the ICU (Gram-positive n = 7, Gram-negative n = 5). Among the 48 patients with non-severe sepsis, 25 patients had Gram-negative etiology, 21 Gram-positive, and 2 were miscellaneous (polymicrobial). Median age in the group of patients with sepsis was 69 years. Sex distribution was female n = 28 (47.5%) male n = 31 (52.2%). Median Charlson comorbidity score was 1.0 (Table 1).

**Table 1:** Characteristics of patients with sepsis, Gram-staining, and HLA-DR expression

	**Sepsis severity**			**Gram-staining**		**Charlson comorbidity score**				**Sex**	
	**Non-severe**	**Severe**	**Shock**	**G+**	**G-**	**0**	**1-2**	**3-4**	**>5**	**Male**	**Female**
**N **(%)	48 (81.4)	8 (13.6)	3 (5.1)	29 (49.2)	27 (45.8)	20 (33.9)	28 (47.4)	8 (13.6)	3 (5.1)	31 (52.5)	28 (47.5)
**mHLA-DR **AB/cell											
Median value	23,891	12,659	10,564*	12,482	30,938	18,606	21,475	29,178	7,398*	18,267	19,764
IQR	12,773-45,370	8,949-21,087		9,262-22,480	18,267-51,908	10,653-57,296	12,985-35,502	11,085-39,176		9,710-40,961	12,571-33,099
**HLA-DRA** ratio											
Median value	3.37	1.47	0.62*	2.12	4.02	2.83	3.16	3.33	0.37*	2.94	2.83
IQR	1.78-5.48	0.95-3.7		0.88-3.64	1.96-6.72	0.98-6.71	1.78-4.81	1.23-4.41		0.93-4.43	1.04-5.29

*Interquartile range (IQR) not possible to count because of small patient numbers.

### Characteristics of blood donors

The median age in the control group was 63 years. Sex distribution was female n = 8 (26.7%) and male n = 22 (73.3%).

### Monocyte surface expression of HLA-DR

**Figure 1:**
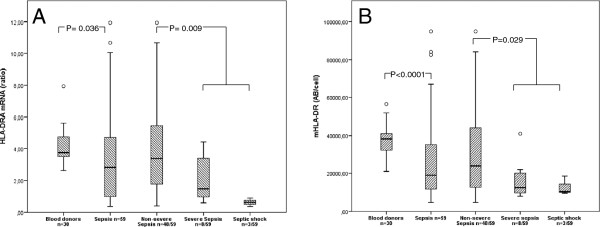
**HLA-DR expression in septic patients with different sepsis severity.** mRNA expression levels **(A)** and monocyte surface expression **(B)** of human leukocyte antigen (HLA)-DR at day 1 or 2 after admission. Statistical differences between septic patients and controls were demonstrated in both HLA-DRA **(A)** (*P* = 0.036) and mHLA-DR **(B)** (*P* <0.0001) expression. Significant differences were demonstrated in HLA-DRA **(A)** (*P* = 0.009) and mHLA-DR **(B)** (*P* = 0.029) expression when the severity groups of non-severe (n = 48) and severe sepsis/septic shock (n = 11) were compared.

In patients with sepsis (n = 59), the median value of mHLA-DR was 19,007 AB/c, and IQR was 11,488 to 36,654 AB/c. In blood donors (n = 30), the median value of mHLA-DR was 38,251 AB/c (IQR 31,997 to 41,113). Statistical analysis demonstrated a significant difference in surface expression between septic patients and controls (*P* <0.0001) (Figure 1).

### HLA-DRA and CIITA mRNA levels in whole blood

In patients with sepsis (n = 59), the median level of HLA-DRA ratio was 2.83 (IQR 0.97 to 4.73). In healthy donors (n = 30), the median of HLA-DRA ratio was 3.75 (IQR 3.46 to 4.77). A statistically significant difference in mRNA expression of HLA-DRA levels was seen between septic patients and healthy donors (*P* = 0.036) (Figure 1). The median level of CIITA ratio in patients with sepsis was 0.14 (IQR 0.06 to 0.21) and was significantly lower than in the control group (*P* <0.0001). Median CIITA level in healthy controls was 0.36 (IQR 0.25 to 0.43).

### Correlations between mRNA expression levels (HLA-DRA, CIITA) and surface expression of HLA-DR

**Figure 2:**
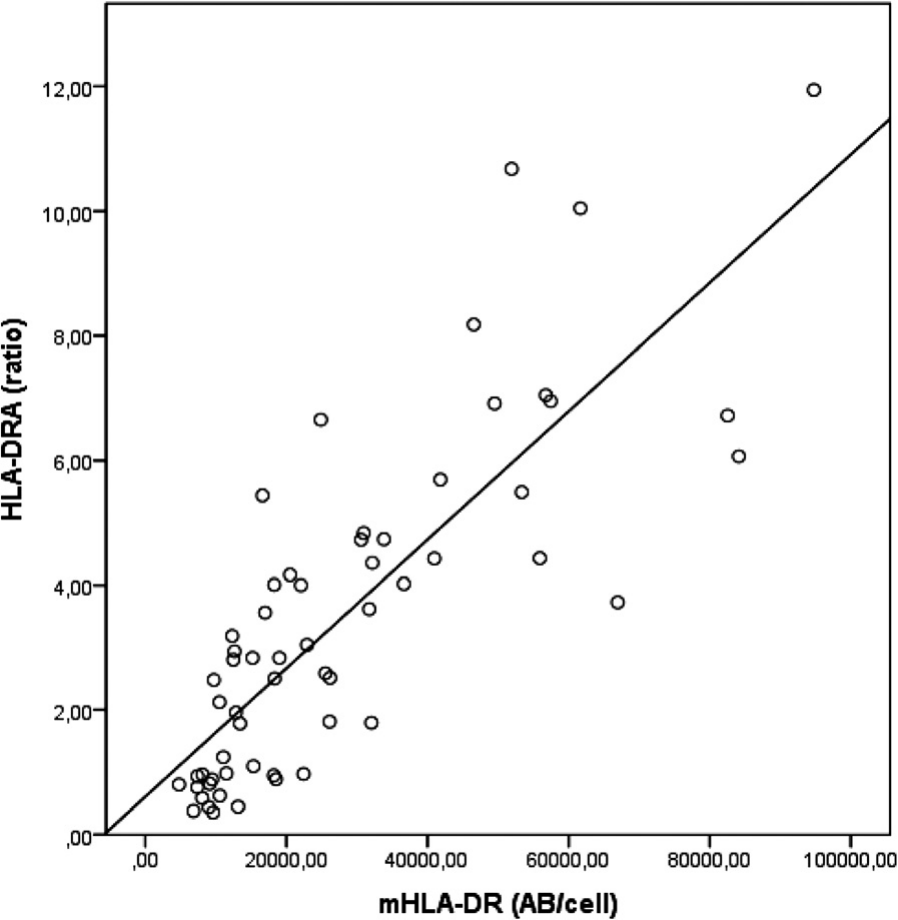
**HLA-DRA in relation to mHLA-DR.** mRNA expression levels of HLA-DRA in whole blood and monocyte surface expression of HLA-DR in 59 patients with sepsis at day 1 or 2 after admission.

In patients with sepsis (n = 59), blood samples were collected simultaneously for HLA-DR monitoring by qRT-PCR (transcripts of HLA-DRA and CIITA) and flow cytometry. A statistically significant strong correlation (*r* = 0.842, *P* <0.0001) was seen between surface mHLA-DR and mRNA levels of HLA-DRA. The relationship between HLA-DRA and mHLA-DR is shown in Figure 2.

### Expression of HLA-DRA and mHLA-DR in sepsis caused by Gram-negative and Gram-positive bacteria

**Figure 3:**
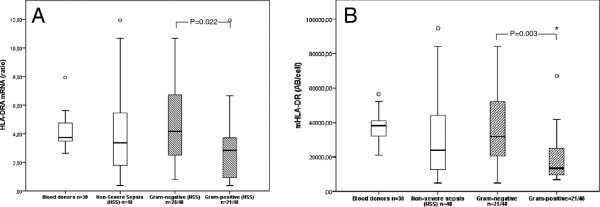
**HLA-DR expressions in Gram-positive and Gram-negative non-severe sepsis.** mRNA expression levels **(A)** and monocyte surface expression **(B)** of human leukocyte antigen (HLA)-DR at day 1 or 2 after admission. Significant differences were demonstrated in both mRNA expression levels of HLA-DRA **(A)** (*P* = 0.022) and monocyte surface expression of HLA-DR **(B)** (*P* = 0.003) when the groups of Gram-positive and etiology were compared.

In non-severe sepsis caused by Gram-positive bacteria (n = 21), HLA-DRA and mHLA-DR expression was significantly lower compared with healthy controls (*P* = 0.006 and *P* <0.0001, respectively). There was no significant difference in HLA-DRA or mHLA-DR expression in non-severe sepsis (n = 24) caused by Gram-negative bacteria compared with controls (*P* = 0.310, *P* = 0.648). Significant differences in HLA-DR expression measured by either qRT-PCR or flow cytometry were observed between the non-severe groups of Gram-positive and Gram-negative etiology (HLA-DRA *P* = 0.022; mHLA-DR *P* = 0.003) (Figure 3).

### Expression of HLA-DRA and mHLA-DR in relation to sepsis severity

There were significant differences in both mHLA-DR and HLA-DRA expression between the two groups of non-severe (n = 48) and severe (n = 11) sepsis/septic shock (mHLA-DR *P* = 0.029, HLA-DRA *P* = 0.009).

## Discussion

To reduce mortality in sepsis, it is important to focus on the late phase of sepsis, representing the period with a significantly higher death rate. Given this, it is likely to believe that the immunosuppressive state, described as a common feature of the late phase of sepsis, plays a pivotal role in the persisting high mortality rate.

In terms of clinical information, it has been suggested to perform mHLA-DR analysis routinely on ICU patients in order to evaluate the immune function [27]. So far, flow cytometry is considered the gold standard in HLA-DR monitoring. However, flow cytometry has important practical limitations: (a) results vary dramatically because of differences in specimen handling, (b) blood samples have to be analyzed immediately and cannot be stored for later analyses, and (c) samples cannot be collected from health-care units without laboratory equipment for flow cytometry. These important limitations of traditional HLA-DR monitoring clearly hamper the clinical usage and feasability of larger multicenter studies required in this field. An mRNA-based approach for HLA-DR expression allows samples to be stored in a frozen state, to be collected at different sites, and to be sent to a collaborating laboratory for later analysis.

In this study, we investigated HLA-DR expression in 59 patients with sepsis, all with blood cultures growing pathogenic bacteria. Patients were included independently of sepsis severity or underlying disease, in contrast to previous studies focusing on HLA-DR in severe sepsis or septic shock [25]. A significantly lower HLA-DR expression in the total number of septic patients compared with controls was observed in both mRNA expression levels of HLA-DRA and CIITA from whole blood and in monocyte surface expression of HLA-DR. A low expression level of mRNA encoding CIITA, the major transactivator of HLA-DR gene expression, represents indirectly a transcriptional downregulation of HLA-DR genes in patients with sepsis [23]. These results confirm the recently presented pathophysiologic mechanism in sepsis HLA-DR expression, suggesting a transcriptional downregulation of a gene panel required for mHLA-DR expression [23,24].

To perform a valid method for quantitation of HLA-DR expression at the mRNA level, it is important to avoid inter-individual variations due to the genetic polymorphism in the HLA-DR genes. Hence, we found it preferable to monitor HLA-DRA, encoding the non-polymorphic region of the alfa-chain of the HLA-DR molecule.

A high correlation between the mRNA expression level of HLA-DRA by qRT-PCR in whole blood and the surface expression of HLA-DR by flow cytometry was demonstrated in this study. The loss of HLA-DR has previously been explained by downregulation of a gene panel essential for mHLA-DR expression, but so far the correlation with mHLA-DR has been studied in only minor patient cohorts [23,24,28]. The different techniques of HLA-DR monitoring in this study correlate highly but are not analogous since we measure the HLA-DR expression at two different levels and in different blood cells. Monitoring of HLA-DRA measures the mRNA expression level in whole blood encoding genetic information important for the molecule structure in all blood cells expressing HLA-DR. In contrast, mHLA-DR monitors the surface expression only on monocytes.

In our study, PPIB was used as the reference gene because of its stability in inflammatory conditions [26]. The choice of reference gene and difference in patient cohort size might explain why our results show a higher correlation coefficient compared with the work by Le Tulzo and colleagues (*r* = 0.764) in which Abelson (ABL) was used as the reference gene [23].

In contrast to Pachot and colleagues [24], we noted an overlap in HLA-DR expression between the septic and healthy patients. This might be explained by our wide inclusion criteria, which also included septic patients with non-severe sepsis. The majority (80%) of patients in the present study were not admitted to the ICU.

The expression levels of HLA-DR in the present study were lower in the groups of severe sepsis and septic shock compared with non-severe sepsis. Because of the small number of patients in the severity groups, statistical significance was reached only when the combined groups of severe sepsis and septic shock were compared with non-severe sepsis. However, variations in HLA-DR expression according to sepsis severity show the similar profile when monitored by either qRT-PCR or flow cytometry.

Differences in HLA-DR expression are seen in Gram-positive and Gram-negative sepsis. While HLA-DR is significantly lower in sepsis caused by Gram-positive bacteria in comparison with blood donors, there is no difference in HLA-DR expression when comparing Gram-negative sepsis and controls, although the patients were scored into the same severity group. This variation is seen when monitoring HLA-DR by cell surface expression as well as at the mRNA expression level.

The reason for possible differences in HLA-DR expression between Gram-positive and Gram-negative sepsis is unknown, and data pointing toward differences are preliminary and should be interpreted carefully due to statistical shortcomings of post-hoc analyses.

The high correlation between mRNA levels of HLA-DRA and surface expression of HLA-DR and similarity in displaying variations according to sepsis severity and Gram-staining results indicates a possible consistency between the two methods. However, some limitations of the present study should be considered before solid conclusions can be drawn. The inclusion criteria consisting of early diagnosed bloodstream-positive sepsis lead to recruitment of patients with a variation in sepsis severity. The majority of patients in the present study group were scored in the non-severe septic group with only slightly depressed HLA-DR expression. Simplified Acute Physiology Score II (SAPS II) and Sequential Organ Failure Assessment (SOFA) score were not registered, because of the low percentage of patients admitted to the ICU. Future studies evaluating the agreement between methods in the late sepsis phase and its relation to the burden of secondary infections will be required to state whether this PCR-guided monitoring of immunosuppression may be preferable as a diagnostic tool.

While flow cytometry requires expensive equipment and work load, there is a rapid development of PCR tests with very little hands-on time [27,29]. If commercial easy-handled qRT-PCR assays for mRNA HLA-DR became available, cellular immune function could more easily be monitored to support management of patients with sepsis.

## Conclusions

Patients with sepsis display a diminished expression of HLA-DR at the monocyte surface as well as in the gene expression at the mRNA level. The mRNA expression level of HLA-DRA monitored by qRT-PCR correlates highly with surface expression of HLA-DR and appears to be a promising future biomarker for evaluation of immunosuppression in sepsis.

## Key messages

• Patients with sepsis display a diminished expression of HLA-DR at the monocyte surface as well as in the gene expression at the mRNA level.

• The mRNA levels of HLA-DRA and the monocyte surface expression of HLA-DR correlate highly in the early sepsis phase.

## Abbreviations

ABL: Abelson; bp: Base pairs; CIITA: Class II transactivator; HLA: Human leukocyte antigen; HLA-DRA: mRNA encoding the alpha chain of human leukocyte antigen-DR; ICU: Intensive care unit; IQR: Interquartile range; mHLA-DR: monocyte human leukocyte antigen-DR surface expression; PPIB: Peptidylpropylisomeras B; qRT-PCR: quantitative real-time polymerase chain reaction.

## Competing interests

The authors declare that they have no competing interests.

## Authors’ contributions

SC, AB, ET, BS, KS, and JK designed the study. SC and KS were responsible for enrolment and data collection. AB, SC, and JK established the PCR methodology. AB carried out the laboratory work with the qRT-PCR. ET established the flow cytometry measurements. SC was responsible for data collection and analysis. JK and SC conceived the study and were responsible for statistical analysis and revision of the manuscript. All authors have been involved in writing of the manuscript and revising it critically for important intellectual content and approved the final version.
